# Comparing Surgical and Conservative Treatment on Achilles Tendon Rupture: A Comprehensive Meta-Analysis of RCTs

**DOI:** 10.3389/fsurg.2021.607743

**Published:** 2021-02-18

**Authors:** Guorong She, Qiang Teng, Jieruo Li, Xiaofei Zheng, Lin Chen, Huige Hou

**Affiliations:** ^1^Department of Sport Medicine, First Affiliated Hospital of Jinan University, Guangzhou, China; ^2^Drug Clinical Trial Institution, First Affiliated Hospital of Jinan University, Guangzhou, China

**Keywords:** Achilles tendon rupture, surgery, conservative, meta-analysis, clinical outcome

## Abstract

**Background:** The Achilles tendon is the strongest tendon in human and is frequently injured, mainly in the young to middle age active population. Increasing incidence of Achilles tendon rupture (ATR) is still reported in several studies. Surgical repair and conservative treatment are two major management strategies widely adopted in ATR patients, but the consensus of the optimal treatment strategy is still debated. We aimed at thoroughly reviewing the ATR topic with additional assessments and performed a most comprehensive meta-analysis of randomized controlled trials (RCTs).

**Method:** We comprehensively searched PubMed, Embase, Cochrane, and ClinicalTrial.gov and retrieved all RCTs comparing surgical and conservative treatment on ATR for further analysis. Two independent reviewers performed data extraction and random effect model was adopted when *I*^2^ > 50%, with data presentation of risk ratio, risk difference, or mean difference and 95% confidence interval.

**Results:** A total of 13 RCTs were included in this meta-analysis. A significant difference was observed in re-rupture, complication rate, adhesion to the underlying tendon, sural nerve injury, and superficial infection. A substantial reduction in re-rupture rate could be observed for surgical treatment while the complication rate was higher compared with conservative treatment.

**Conclusion:** Surgical treatment revealed significance in reducing the re-rupture rate but was associated with a higher complication rate, while conservative treatment showed similar outcomes with a lower complication rate. Collectively, we recommend conservative treatment if patients' status and expectations are suitable, but surgeon and physician discretion is also crucial in decision making.

## Introduction

Achilles tendon (AT), a combination of the tendinous portion of gastrocnemius and soleus muscles to form the strongest tendon in the human body, is frequently injured mainly in the young to middle age active population of society, with the average age ranging from 37 to 44 years (1, 2). Increasing incidence of Achilles tendon rupture (ATR) is still reported in several studies due to the increasing older active population in society and male patients are more common than female patients even though a higher rupture force is required in the male (2–4).

The etiology of ATR is rarely discussed while several risk factors are accounted for, such as steroid injection, rheumatoid arthritis, intake of fluoroquinolones, and long-term dialysis (5, 6). For the sake of its specificity in ambulation and activity, appropriate management of ATR is essential.

Surgical repair and conservative treatment are two major management strategies widely adopted in ATR patients, but the consensus of the optimal treatment strategy is still debated. Several previous systemic reviews reported that similar results occurred in surgical or non-surgical treatment with the measurement of clinical score and patient satisfaction (7, 8). In former studies comparing surgical and non-surgical processes, re-rupture rate was the predominant outcome measure to assess the treatment success, while it is relatively low with the current treatment protocols (9–11). Collectively, fully restored function to the former level and self-satisfaction from patients should be taken into consideration as an additional assessment.

Although two previous meta-analyses compared the surgical and non-surgical treatment in ATR, neither of them considered the abovementioned restored function to the former level or self-satisfaction. Moreover, the situation of inadequate inclusion of studies focusing on related topic occurred in both studies. Consequently, we aimed at thoroughly reviewing the ATR topic with additional assessments and performed a most comprehensive meta-analysis of randomized controlled trials (RCTs).

## Methods

### Protocol

This meta-analysis was conducted and performed under the instruction of Meta-analysis Of Observational Studies in Epidemiology (MOOSE) and the Preferred Reporting Items for Systemic Reviews and Meta-Analyses (PRISMA) checklists (12–14).

### Searching Query and Eligibility Criteria

We thoroughly searched online public databases, namely, PubMed, Embase, Cochrane Central Register of Controlled Trials (CENTRAL), and ClinicalTrials.gov, until 1st July 2020 with the keywords of Achilles tendon and surgery with their corresponding MeSH terms. We retrieved all studies comparing surgical vs. conservative treatment in ATR patients for further review. Duplicate studies were excluded, and two authors independently completed the initial title and abstract screening. Only RCTs that reported on the comparison of surgical vs. conservative treatment of ATR were included in this meta-analysis.

After initial title and abstract screening, two independent authors reviewed all retrieved articles with full text. We excluded reviews, letters, editorial comments, conference abstract, discussion, notes, viewpoint, no published full text, and case reports. Delayed treatment for more than 4 weeks was excluded and the same for treatment of re-rupture of ATR. There was no weight bearing or functional rehabilitation protocol restriction. The eligibility criteria were patients with ATR, surgical treatment (open or minimally invasive surgery) vs. conservative treatment (cast immobilization or functional bracing), age >16 years old, treatment initiated within 4 weeks of injury, reporting of re-rupture, complications, functional outcomes, and patients' satisfaction on corresponding treatment and outcomes. Any disagreement on study inclusion was resolved by consensus or routine meeting of all authors listed in this meta-analysis. Detailed information about the eligibility criteria is shown in Table 1.

**Table 1:** Eligibility criteria applied in this meta-analysis.

	**Inclusion criteria**	**Exclusion criteria**
Study type	Only randomized controlled trials.	1. Unfinished studies, unpublished data or no published full text. 2. Reviews, editorials, letters, notes, discussions, comments, conference abstracts etc.
Participants	Involved patients should conform to the following criteria: 1. Achilles tendon rupture within 4 weeks. 2. Age >16-year-old. 3. Adopting comparison on surgical (open or minimally invasive surgery) vs. conservative treatments (cast immobilization or functional bracing).	Non-human subjects
Intervention	Open or minimally invasive surgery.	N/A
Control	Non-surgical treatment (cast immobilization or functional bracing).	N/A
Outcome	1. Re-rupture. 2. Major or minor complication. 3. Functional outcomes. 4. Patients' satisfaction on corresponding treatment and outcomes.	Unpublished data

### Data Extraction

Two authors independently extracted both baseline demographics with all outcomes data, and disagreements were resolved by discussion in a routine meeting to prevent the occurrence of test-qualified pooling (15). All baseline demographics data were extracted from included studies and intersection was obtained for providing more detailed information as far as possible. Author names, country, age, gender, time between injury and treatment, surgical technique, and follow-up were essential elements to extract. The same strategy was administered in outcome data extraction in order to make the most comprehensive pooled analysis.

### Primary and Secondary Outcome

Regarding raised concerns about recent studies (9–11), different from the previous meta-analysis, return to sport (the same level as pre-treatment) and re-rupture rate were adopted as primary outcomes. Secondary outcomes consisted of complication rate (defined as complication occurred after treatment except for re-rupture), deep vein thrombosis, adhesion of scar to the underlying tendon, sural nerve injury, superficial infection, deep infection, period of absence from work, functional scores of Achilles Tendon Rupture Score (ATRS) (16), and mean of dorsiflexion and plantarflexion. In addition, in scenario of returning to sport, patients who recover to the same level as pre-treatment was pooled, which might describe the efficacy of treatment distinctly. Combined results were pooled in studies that reported open as well as minimally invasive surgery.

### Assessment of Heterogeneity

We analyzed statistical heterogeneity between studies by means of *I*^2^-test and, the criteria were *I*^2^ > 50% for existence of heterogeneity and *I*^2^ > 70% for high heterogeneity (17).

### Risk of Bias Assessment

Two authors independently assessed the risk of bias from each study under the instruction of Cochrane Risk of Bias Tool, and the same was done for protocols of included studies (18). During the entire assessment process, selection bias, performance bias, attrition bias, and reporting bias were analyzed, and publication bias was evaluated as well as visualization via Egger's-test (19). Collectively, risk of bias summary graph and funnel plot would be used to review bias existence better.

### Statistical Analysis

All procedures involved in this meta-analysis were performed under Revman (Version 5.3). Both continuous and dichotomous variables were presented in this study. Continuous variables were presented as mean with standard deviation and other forms of data presentation would be converted using the instruction described in the Cochrane Handbook for Systematic Reviews of Interventions and several methods reported in previous studies (20–24). Dichotomous variables were presented as events and the total number of events. The Mantel–Haenszel model was used to analyze the pooled outcomes with the presentation of the risk ratio (RR) and 95% confidence interval (CI). A fixed model would be adopted when *I*^2^ <50% while the random effect model was employed once *I*^2^ > 50%. We administered overall the effect *Z*-test to determine the significance level for pooled effects. For the stratified analysis, a test for subgroup differences was used to determine the significant level. We set the significance level as a *P*-value lower than 0.05.

## Results

### Literature Search

**Figure 1:**
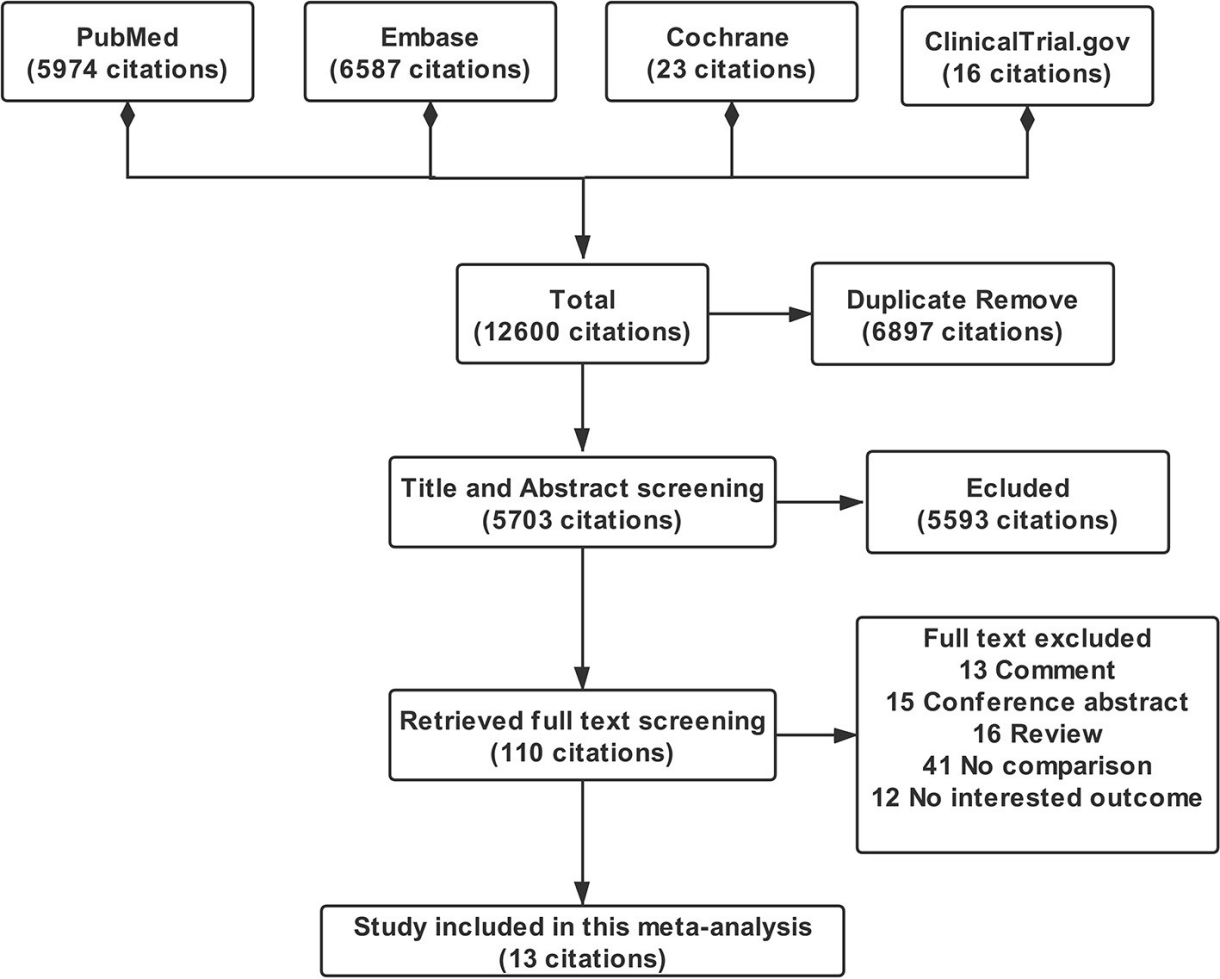
PRISMA flowchart of this meta-analysis.

All literature screening processes were performed with Endnote X8. After literature searching, a total of 5,974 citations from PubMed, 6,587 citations from Embase, 23 citations from Cochrane, and 18 citations from Clinicaltrial.gov were obtained. We excluded 6,897 duplicate citations by using Endnote duplicate citations finding function. After initial title and abstract screening, 5,593 citations were excluded and disagreement would be resolved by the routine meeting of the research group. During full text screening, a total of 97 citations not compliant with the criteria were excluded and 13 citations of studies were included in this meta-analysis eventually (11, 25–36). The PRISMA flowchart of this meta-analysis is displayed in Figure 1.

### Baseline Characteristics

A total of 1,164 patients were included in this study, with 603 patients in the surgical group and 561 patients in the non-surgical group. The mean age of enrolled patients was around 40 years old, ranging from 18 to 63 years old, which conformed to the regular ATR population. Overall, male and female patients consisted of 84 and 16% of the population included in the study. For the time period between injury and treatment, 2 days was the shortest period reported by Twaddle et al. (29) while 21 days was the longest period reported by Nistor et al. (25). In addition, each included studies' surgical techniques were extracted for better interpretation of baseline characteristics, and end-to-end Bunnell type was the most adopted technique for ATR repair. Last but not least, different follow-up periods could be a significant factor affecting the results so that it was recorded. One and two years were the widely accepted follow-up period among the included RCTs. The detailed information of baseline characteristics of each RCTs is shown in Table 2.

**Table 2:** Baseline characteristics of included studies.

**References**	**Country**	**Patients**			**Age**		**Gender (M/F)**		**Time between injury and treatment (days)**	**Surgical technique**	**Follow-up (months)**	
		**Overall no**	**OP**	**NON**	**OP**	**NON**	**OP**	**NON**			**OP**	**NON**
Nistor (25)	Sweden	107	49	51	41 (21–77)		96/11		21 days or less	Bunnell-type with coaptation sutures to close the paratendon	30 (12–60)	
Cetti et al. (26)	Denmark	111	56	55	41.2 (27–59)	37.8 (21–65)	47/9	45/10	7 days or less	End to end, Bunnell-type suture	12	12
Möller et al. (27)	Sweden	112	59	53	39.6 (21–63)	38.5 (26–59)	51/8	48/5	7 days or less	End to end, modified Kessler	24	24
Costa et al. (28)	United Kindom	96	48	48	42 (28–61)	42 (29–69)	18/4	22/3	7 days or less	End to end, augmented repair	12	12
Twaddle and Poon (29)	New Zealand	50	25	25	41.8	40.3	14/6	14/8	2 days or less	End to end, Krackow-type stitch	12	12
Metz et al. (30)	Netherlands	83	42	41	40 (23–63)	41 (25–62)	31/11	35/6	3 days or less	Bunnell-type suture in proximal tendon, through lateral aspect of calcaneus distally	12	12
Nilsson-Helander et al. (31)	Sweden	97	49	48	40.9 (8.8)	41.2 (9.5)	40/9	39/9	3 days or less	End to end, modified Kessler	12	12
Willits et al. (32)	Canda	144	72	72	39.7 (11)	41.1 (8.0)	59/13	59/13	14 days or less	End to end, Krackow-type stitch	24	24
Keating and Will (33)	United Kindom	80	39	41	41.2 (27–59)	39.5 (21–58)	28/11	32/9	10 days or less	End to end, Kessler stitch, interrupted circumferential stitch	12	12
Olsson et al. (11)	Sweden	100	49	51	39.8 (8.9)	39.5 (9.7)	39/10	47/4	4 days or less	End to end, Mmodified Kessler, epitendnous cross-stitch	12	12
Lantto et al. (34)	Finland	60	32	28	40 (27–57)	39 (28–60)	30/2	25/3	7 days or less	End-to-end open repair	18	18
Manent et al. (35)	Spain	34	23	11	41 (18–51)	42 (26–51)	10/1	21/2	10 days or less	Percutaneous repair/ Double Bunnell suture	12	12
Fischer et al. (36)	Germany	90	60	30	39.5 (21–58)	45.2 (25–60)	27/3	54/6	N/A	Already established protocol	24	24

*Values are presented with mean (sd or range)*.

*OP, Operative group; NON, non-operative group*.

### Risk of Bias Assessment

Two independent authors strictly assessed the risk of bias across studies under the instruction of Cochrane Collaboration Tool and the visualization of results is displayed in Supplementary Figure 1 (17). Risk of bias was relatively low owing to the characteristics of RCTs. However, an assessment of unclear risk occurred in several studies. Regarding selection bias about random sequence generation, Moller et al. (27) and Keating et al. (33) did not clearly state the situation and unclear risk was assessed in Nistor et al. (25), Fischer et al. (36), and Cetti et al. (26). When it comes to blinding of participants and personnel in performance bias, unclear risk occurred in Nistor et al. and Cetti et al., while high risk was assessed in Fischer et al. Inadequate blinding of assessment was not clearly declared in Nistor et al. and Fischer et al. so that unclear risk was obtained.

Publication bias was assessed by administrating Revman software and Egger's test was adopted. Each outcome measure was assessed individually and visualization of results is shown in Supplementary Figures 2A–L. Inspection of symmetry was obtained, indicating no publication bias among each outcome measure.

### Primary Outcomes

#### Re-rupture Rate

**Figure 2:**
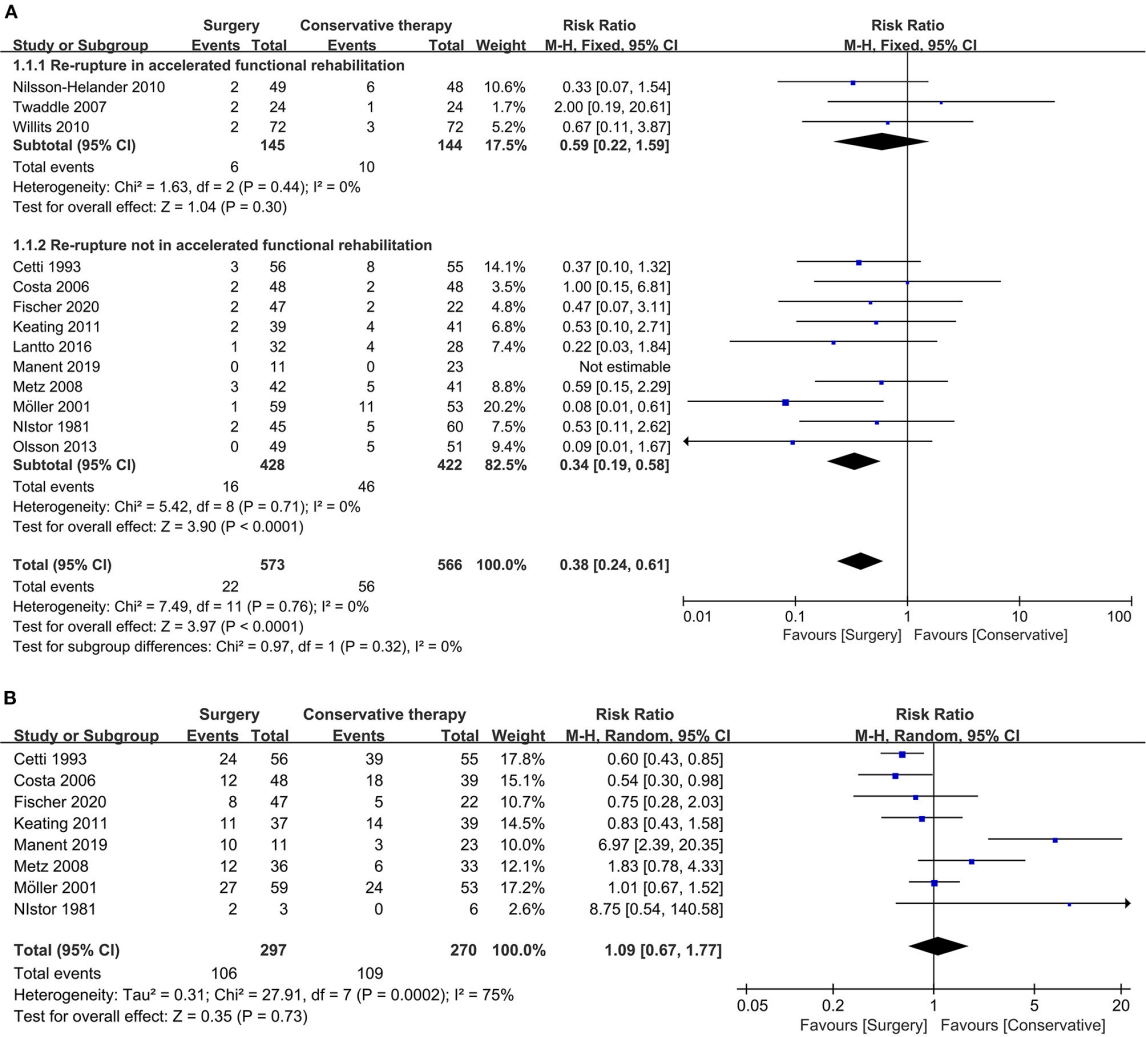
Forest plot of primary outcome measure. **(A)** Forest plot of re-rupture rate. **(B)** Forest plot of return to sport (same level).

All included 13 studies reported the result of re-rupture rate, and we divided it into re-rupture in accelerated functional rehabilitation and re-rupture not in accelerated functional rehabilitation as subgroup analysis. In the subgroup of re-rupture that occurred in accelerated functional rehabilitation, no significant difference between surgical and conservative treatment could be observed (three studies, 289 participants, *Z* = 1.04, *P* = 0.30, *I*^2^ = 0%, RR: 0.59, 95% CI: 0.22 to 1.59). In contrast, compared with the conservative group, significant reduction in re-rupture rate not in accelerated functional rehabilitation could be observed in the surgical treatment group (10 studies, 850 participants, *Z* = 3.90, *P* < 0.0001, *I*^2^ = 0%, RR: 0.34, 95% CI: 0.19 to 0.58). Collectively, the overall result showed that surgical treatment was associated with significant reduction in re-rupture rate (13 studies, 1139 participants, *Z* = 3.97, *P* < 0.0001, *I*^2^ = 0%, RR: 0.38, 95% CI: 0.24 to 0.41). Detailed information about the re-rupture rate is shown in Figure 2A.

#### Return to Sport

There were eight studies that reported the result of return to sport among patients receiving ATR repair. Cetti et al. (26) and Costa et al. (28) reported the favorable outcome of surgical treatment in recovering ATR patients' sporting capacity compared with conservative management, while Manent et al. (35) reported the opposite result favoring conservative treatment. Collectively, the overall result indicated no significant difference between surgical and conservative treatment in sport capacity recovery (eight studies, 567 participants, *Z* = 0.35, *P* = 0.73, *I*^2^ = 75%, RR: 1.09, 95% CI: 0.67 to 1.77). Detailed information about returning to sport is shown in Figure 2B.

### Secondary Outcomes

#### Complication Rate

**Figure 3:**
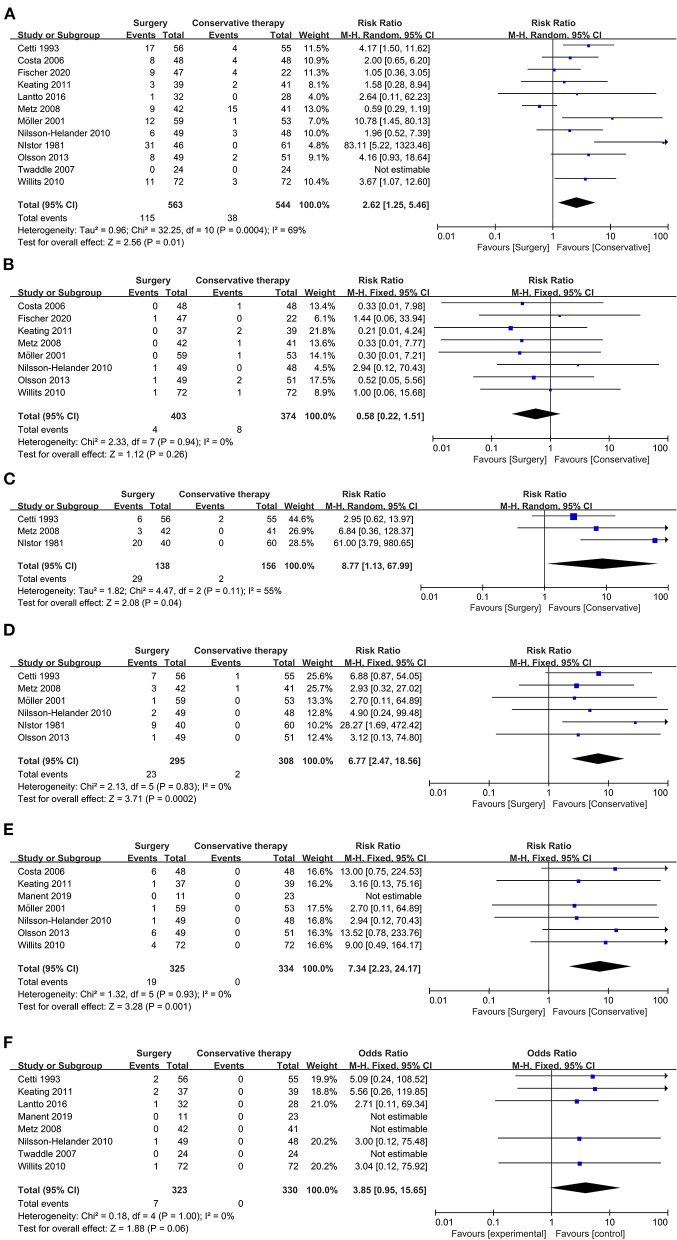
Forest plot of secondary outcome measure indicating complication. **(A)** Forest plot of complication rate. **(B)** Forest plot of deep vein thrombosis. **(C)** Forest plot of adhesion to underlying tendon. **(D)** Forest plot of sural nerve injury. **(E)** Forest plot of superficial infection. **(F)** Forest plot of deep infection.

We defined complication rate as complication that occurred after ATR treatment other than re-rupture, and it was reported in 12 of the included studies. The overall result indicated that the complication rate after treatment in the conservative treatment group was significantly lower than that in surgical treatment group (12 studies, 1,107 participants, *Z* = 2.56, *P* = 0.01, *I*^2^ = 69%, RR: 2.62, 95% CI: 1.25 to 5.46). Main complications that occurred after ATR treatment were deep vein thrombosis, adhesion of scar to the underlying tendon, the sural nerve injury, and superficial and deep infection. Detailed information about the overall complication rate is shown in Figure 3A.

#### Deep Vein Thrombosis

Deep vein thrombosis, a severe complication that usually occurred after ATR treatment owing to plaster casting immobilization (28), was reported in eight of the included studies. The overall result showed that no significant evidence could be obtained to distinguish better management strategy to avoid deep vein thrombosis (eight studies, 777 participants, *Z* = 1.12, *P* = 0.26, *I*^2^ = 0%, RR: 0.58, 95% CI: 0.22 to 1.51). Detailed information about deep vein thrombosis is displayed in Figure 3B.

#### Adhesion

Adhesion of scar to the underlying tendon was reported in three of the included studies, and it might lead to secondary surgery. The overall result revealed that the surgical process might increase the incidence of adhesion of scar to underlying tendons (three studies, 294 participants, *Z* = 2.08, *P* = 0.04, *I*^2^ = 55%, RR: 8.77, 95% CI: 1.13 to 67.99). Detailed information about the adhesion of scar to the underlying tendon is displayed in Figure 3C.

#### Sural Nerve Injury

Disturbance in sensation of ATR patients after treatment due to sural nerve injury was reported in six of the included studies. The overall results showed that a significantly increased incidence of sural nerve injury occurred in patients with surgical treatment than conservative management in ATR (six studies, 603 participants, *Z* = 3.71, *P* = 0.0002, *I*^2^ = 0%, RR:6.77, 95% CI: 2.47 to 18.56). Detailed information is shown in Figure 3D.

#### Infection

Wound infection was a common complication of surgical treatment in ATR repair, and it could be divided into superficial and deep infection. For superficial infection, compared with the surgical treatment group, conservative management showed significant evidence to prevent infection after treatment (seven studies, 659 participants, *Z* = 3.28, *P* = 0.001, *I*^2^ = 0%, RR: 7.34, 95% CI: 2.23 to 24.17). Detailed information is shown in Figure 3E.

In contrast, regarding deep infection, there was no significant difference between surgical treatment and conservative treatment group, even though no case of deep infection in the conservative group was reported (eight studies, 653 participants, *Z* = 1.88, *P* = 0.06, *I*^2^ = 0%, RR: 3.85, 95% CI: 0.95 to 15.65). Detailed information about deep infection is shown in Figure 3F.

#### Period of Absence From Work

**Figure 4:**
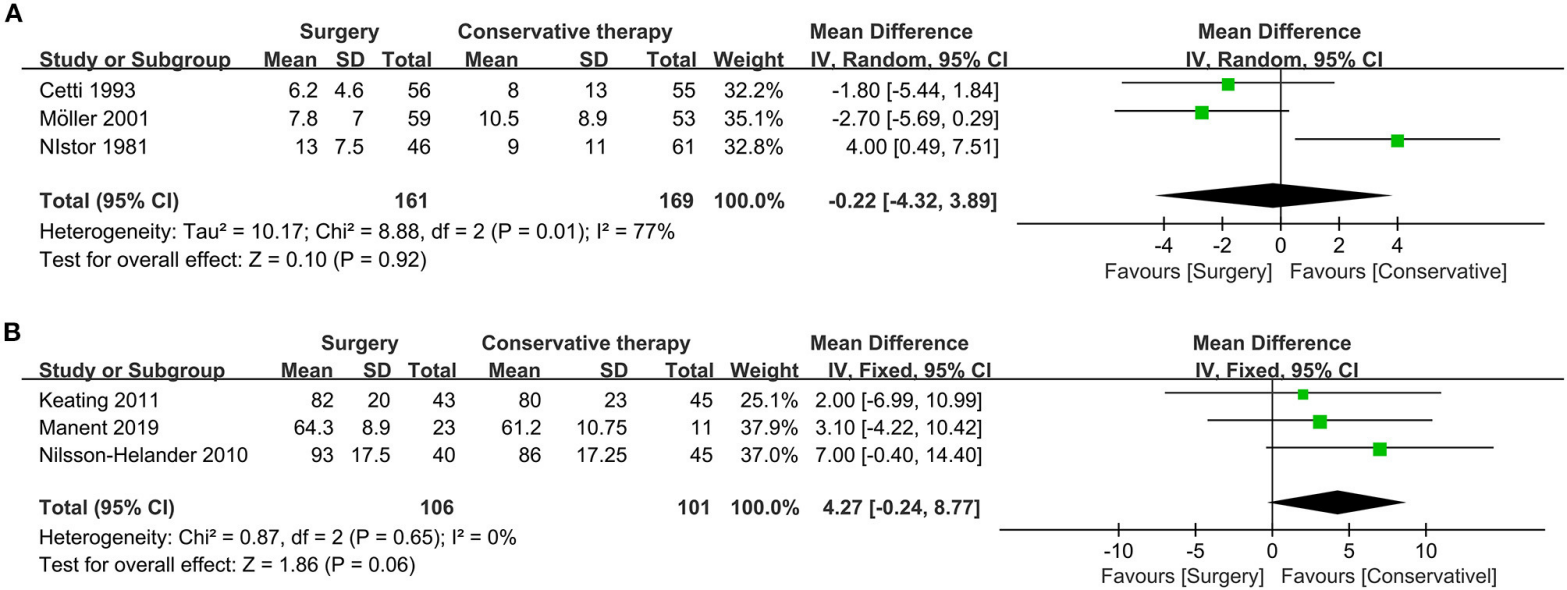
Forest plot of secondary outcome measure indicating period of absence from work and ATRS. **(A)** Forest plot of period of absence from work. **(B)** Forest plot of functional score (ATRS).

ATR results in loss of motor ability as well as absence from patients' occupation so that a different time period is an essential assessment index. The pooled result showed that neither surgical treatment nor conservative management had a shorter period of absence from work (three studies, 330 participants, *Z* = 0.10, *P* = 0.92, *I*^2^ = 77%, RR: −0.22, 95% CI: −4.32 to 3.89). Detailed information is displayed in Figure 4A.

#### ATRS Functional Score

ATRS functional score, with high reliability, validity, and sensitivity for quantifying functional outcome of patient receiving ATR treatment, is an indispensable index to determine the better treatment (16). According to pooled results, there was no significant difference between surgical and conservative treatment regarding ATRS assessment (three studies, 207 participants, *Z* = 1.86, *P* = 0.06, *I*^2^ = 0%, RR: 4.27, 95% CI: −0.24 to 8.77). Detailed information about the ATRS assessment is displayed in Figure 4B.

#### Flexion

**Figure 5:**
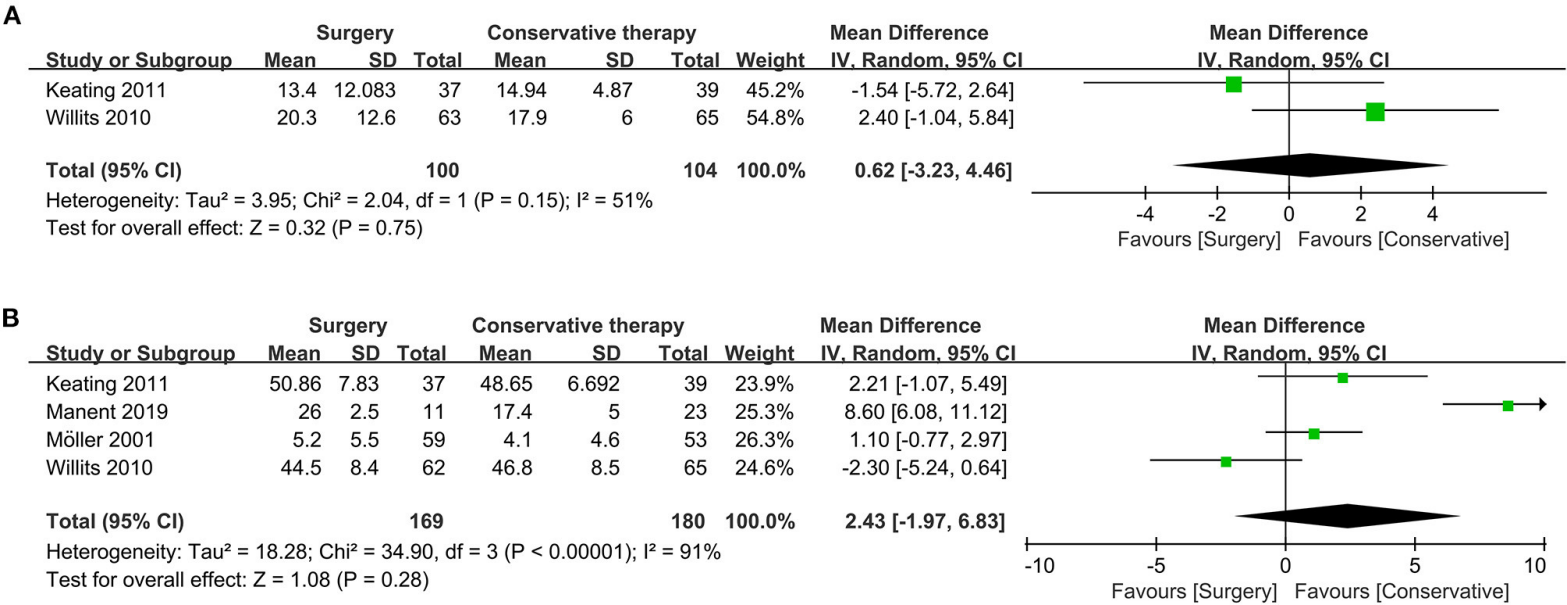
Forest plot of secondary outcome measure indicating flexion. **(A)** Forest plot indicating mean of dorsiflexion. **(B)** Forest plot indicating mean of plantarflexion.

Range of motion is a reflection of joint motor ability, and dorsiflexion and plantarflexion are suitable indexes to the assessment. For mean dorsiflexion, the surgical treatment group was similar to the conservative treatment group (two studies, 204 participants, *Z* = 0.32, *P* = 0.75, *I*^2^ = 51%, RR: 0.62, 95% CI: −3.23 to 4.46). Detailed information about dorsiflexion is shown in Figure 5A.

Similarly, no significant difference could be observed regarding the pooled result of mean plantarflexion (four studies, 349 participants, *Z* = 1.08, *P* = 0.28, *I*^2^ = 92%, RR: 2.43, 95% CI: −1.97 to 6.83). Detailed information about plantarflexion is shown in Figure 5B.

## Discussion

### Innovation

This is the most comprehensive meta-analysis of RCTs comparing outcomes after receiving surgical treatment vs. conservative treatment of ATR. The overall results revealed that surgical treatment had a lower re-rupture rate, while no significant difference was found in the subgroup of accelerated functional rehabilitation with early range of motion, which might indicate that early involvement of rehabilitation was not beneficial to functional recovery. In addition, for pooled results of return to sport, which is first treated as primary outcome, no significant difference could be obtained. In contrast, conservative treatment was associated with a lower complication rate (other than re-rupture), which should be taken into consideration when deciding on treatment.

In comparison with the former meta-analysis of this topic performed by Deng et al. (37), five more studies are included in this meta-analysis, which makes it the most comprehensive one. Deng et al. have taken re-rupture rate, deep vein thrombosis, return to sport, ankle range of motion, and related score into consideration, while adhesion, sural nerve injury, period of absence from work, and infection are added in our study. Moreover, primary outcomes and secondary outcomes are separated in this meta-analysis, which clearly provides different levels of evidence for clinical practice. Collectively, with more included studies and additional pooled outcomes, concluded evidences are solid.

To the best of our knowledge, treatment on ATR should be focused on optimal functional recovery accompanied by the least complication. Combined with the novel primary outcome of return to sport included in this study, similar functional recovery was observed between surgical and conservative treatment group, even though surgical treatment was recommended owing to a lower re-rupture rate according to previous studies (38, 39). Moreover, conservative management accompanied with early weight bearing of the injured tendon was reported to stimulate collagen and healing process, leading to a similar re-rupture rate as surgical treatment (10, 40–42). Collectively, a novel recommendation of more than just considering re-rupture and conservative treatment with similar functional recovery as well as a lesser complication on ATR might be considered if patients' status were suitable.

### Exploration of Complications

Till now, the optimal treatment for ATR patients is mainly based on expert consensus and on the basis of judgment from the chief clinician. Surgical repair, with a lower re-rupture rate, is favorable in most cases, while complications (20.4 vs. 7.0%) other than re-rupture are troublesome such as deep vein thrombosis, wound infection, and sensation disturbance (sural nerve injury).

Incidence of deep vein thrombosis is reported from 0.3–50%, and it is a significant factor causing poor quality of life as well as the burden of social cost (43–46). Immobilization such as plaster casting has been a potential pathogenesis leading to deep vein thrombosis (47). Although pooled results of deep vein thrombosis did not reveal a significant difference, it seemed to occur frequently in the conservative group (2.1%), which might be attributable to a long period of plaster casting. Consequently, thromboprophylaxis is necessary after ATR treatment and intermittent pneumatic compression has been reported to be highly effective in reducing deep vein thrombosis in ATR patients (43).

Wound infection, classified as superficial and deep infection, is one of the major complications in ATR patients receiving surgical repair. In our study, superficial (5.8%) and deep infection (2.2%) were reported in the surgical treatment group, which was deleterious and intractable with poor outcome (48). A recent meta-analysis has concluded that a minimally invasive method could significantly reduce wound infection rate compared with open surgery (49). Furthermore, negative pressure wound therapy has been reported to be effective for post-operative wound infection of ATR, which could be adopted (50).

Regarding sural nerve injury, leading to sensational disturbance after treatment, the incidence in surgical treatment (7.8%) was significantly higher than conservative treatment. Direct damage in open repair or lack of visualization in minimally invasive operative procedures has been the potential reason for causing injury and a modified medialization of percutaneous suture was reported with a lower incidence of sural nerve injury (51).

### Exploration of Functional Outcome

Functional outcomes were defined as period of absence from work and ATRS score, and the pooled result revealed a similar outcome between the surgical and conservative treatment group. Not surprisingly, similar results were found in pooled outcomes of mean dorsiflexion and plantarflexion. Collectively, in functional recovery, conservative treatment might have a similar prognosis to surgical repair.

### Limitation and Implication for Future Research

Although a total of 13 RCTs are included in this meta-analysis, the recorded categories of complications are still limited, which results in disturbance of comprehensive assessment of each treatment strategy. Specifically, for deep vein thrombosis, more cases occurred in the conservative treatment group, but thromboprophylaxis is only reported in four included studies (11, 27, 31, 33), which may confuse the result. Functional outcomes are similar in both groups according to our study's pooled result, but the number of studies reporting functional outcomes such as period of absence from work, ATRS score, and dorsiflexion and plantarflexion are limited. Furthermore, different periods of follow-up, surgical techniques and conservative management strategy may lead to different outcomes.

Future research with a specific focus on comorbidities other than re-rupture is necessary, and it will provide more clues for surgeons or physicians to make an optimal decision. Regarding the summary of our results, a novel inspiration about adopting conservative management as the major treatment plan with lesser complication and similar outcome has been generated. However, patients' expectations are also essential that the athletic population may prefer surgical treatment to expedite recovery and prolong their professional careers (52). Consequently, future RCTs are needed to investigate if surgical and conservative treatment have similar outcomes and prognosis, especially in return-to-sport ability.

## Conclusion

In this meta-analysis, surgical treatment was revealed to be significant in the reduction of re-rupture rate but associated with a higher complication rate. Conservative treatment was found to be capable of having similar functional outcomes with surgical treatment. Collectively, we recommend conservative treatment if patients' status and expectations are suitable, but surgeon and physician discretion is also important in decision making.

## Data Availability Statement

The original contributions generated in the study are included in the article/Supplementary Material, further inquiries can be directed to the corresponding authors.

## Author Contributions

GS and QT: conceptualization, literature researching, methodology, data analysis, and manuscript writing. JL and XZ: investigation. LC and HH: supervision, conceptualization, professional suggestion, and revision. All authors have read and approved the manuscript.

## Conflict of Interest

The authors declare that the research was conducted in the absence of any commercial or financial relationships that could be construed as a potential conflict of interest.

## Supplementary Material

Supplementary Figure 1 Visualization of bias assessment. **(A)** Summary of risk of bias. **(B)** Risk of bias graph.

Supplementary Figure 2 Funnel-plot indicating publication bias of each pooled outcome. **(A)** Re-rupture rate; **(B)** Return-to-sport (Same level); **(C)** Complication rate; **(D)** Deep vein thrombosis; **(E)** Adhesion to underlying tendon; **(F)** Sural nerve injury; **(G)** Superficial infection; **(H)** Deep infection; **(I)** Period absence from work; **(J)** ATRS; **(K)** mean of dorsiflexion; **(L)** mean of plantarflexion.
