# Supplementary figures and images for: Comparing Surgical and Conservative Treatment on Achilles Tendon Rupture: A Comprehensive Meta-Analysis of RCTs
Source: Front Surg. 2021 Feb 18;8:607743. doi: 10.3389/fsurg.2021.607743 (PMC7931800; doi:10.3389/fsurg.2021.607743)

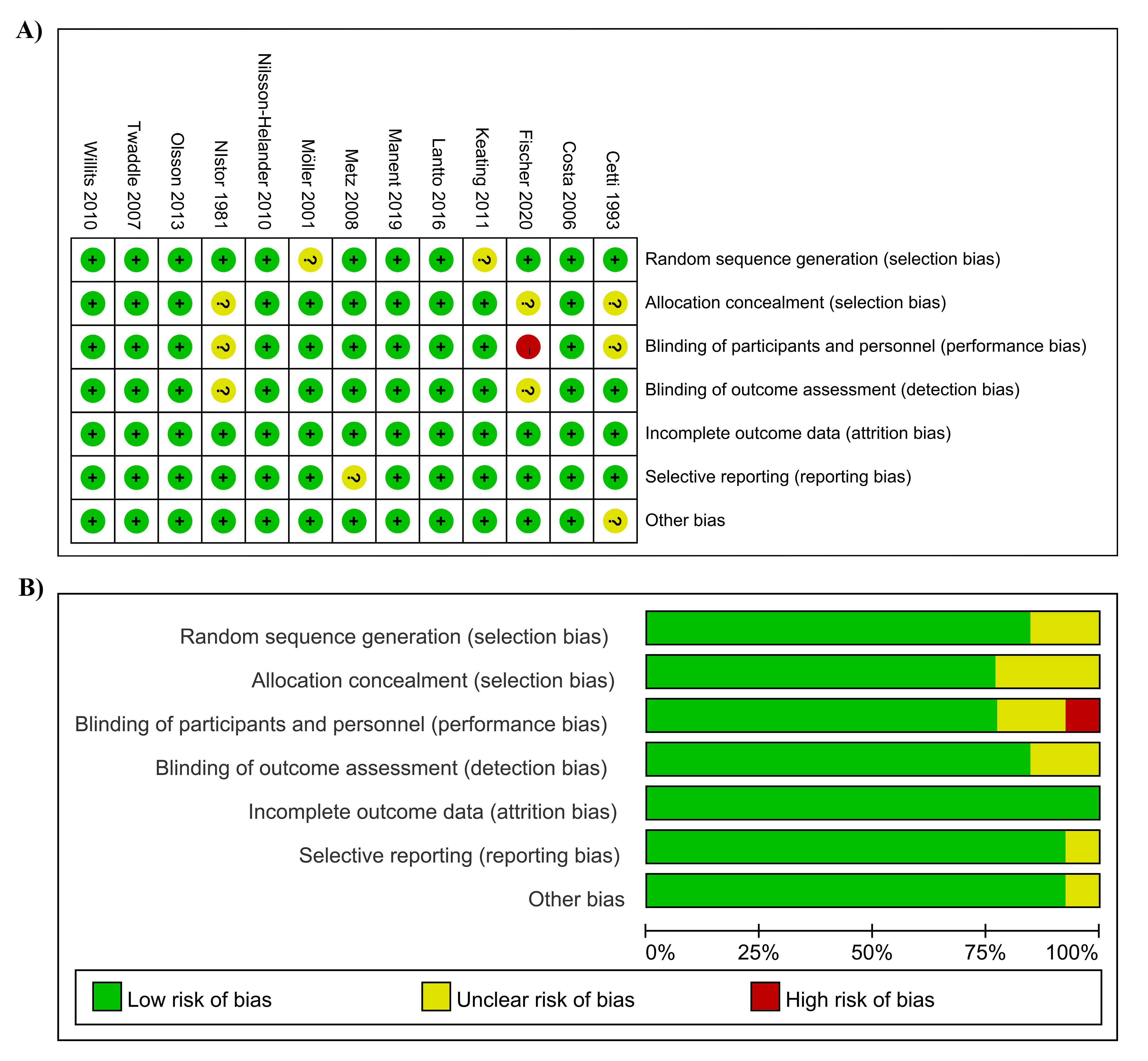

Supplement: Supplementary Figure 1 — Visualization of bias assessment. (A) Summary of risk of bias. (B) Risk of bias graph. [file Image_1.JPEG]
